# BILIARY FISTULAS ASSOCIATED WITH LIVER TRANSPLANTATION VERSUS OTHER ETIOLOGIES: WHAT IS THE SUCCESS RATE OF THE ENDOSCOPIC TREATMENT?

**DOI:** 10.1590/0102-672020220002e1685

**Published:** 2022-09-16

**Authors:** Leonardo MARINHO, Fred Olavo Andrade Aragão CARNEIRO, Leonardo José Sales COSTA, José Huygens Parente GARCIA, Miguel Ângelo NOBRE-E-SOUZA, Marcellus Henrique Loiola Ponte de SOUZA

**Affiliations:** 1 Walter Cantídio University Hospital, Endoscopy Unit - Fortaleza (CE), Brazil;; 2 Universidade Federal do Ceará, School of Medicine, Department of Clinical Medicine - Fortaleza (CE), Brazil;; 3 Universidade Federal do Ceará, School of Medicine, Department of Surgery - Fortaleza (CE), Brazil.

**Keywords:** Biliary Fistula, Liver Transplantation, Cholangiopancreatography, Endoscopic Retrograde, Fístula Biliar, Transplante de Fígado, Colangiopancreatografia Retrógrada Endoscópica

## Abstract

Biliary fistulas typically occur as surgical complications after laparoscopic cholecystectomy, liver transplantation, or partial liver resection.

**AIMS:**

This study aimed to evaluate the efficacy of the endoscopic treatment of biliary fistulae secondary to liver transplantation compared to that of other etiologies.

**METHODS:**

A retrospective study of 25 patients undergoing endoscopic retrograde cholangiopancreatography for biliary fistula from 2015 to 2021 was conducted at the Endoscospy Unit of Walter Cantídio University Hospital. Clinical characteristics and endoscopic success rates of the post-liver transplantation group were analyzed in comparison with those of other etiologies.

**RESULTS:**

The main causes of biliary fistula were liver transplantation (44%) and cholecystectomy complications (44%). The post-liver transplantation group had a significantly higher proportion of male sex (liver transplantation=81.8%, others=28.6%) and older age (liver transplantation=54.1 years, others=42.0 years) and a higher incidence of biliary stenosis (liver transplantation=90.9%, others=14.3%) than those of the group with other etiologies (p<0.05). The two groups received similar treatment types, among which sphincterotomy associated with biliary stent placement was most commonly used. Endoscopic therapeutic success rates showed no significant difference between the post-liver transplantation group (63.6%) and the group with other etiologies (71.4%).

**CONCLUSIONS:**

The endoscopic treatment of biliary fistulae secondary to liver transplantation presented a recovery rate similar to that of other etiologies despite the patients older age and the presence of biliary stenosis

## RESUMO - RACIONAL

As fístulas biliares geralmente ocorrem como complicações cirúrgicas, especialmente após colecistectomia laparoscópica, transplante hepático ou ressecção hepática parcial.

**OBJETIVOS:**

Avaliar a eficácia do tratamento endoscópico das fístulas biliares secundária ao transplante hepático em comparação com outras etiologias.

**MÉTODOS:**

Estudo retrospectivo de 25 pacientes submetidos a Colangiopancreatografia Retrógada Endoscópica por fístula biliar entre 2015 e 2021 no Serviço de Endoscopia do Hospital Universitário Walter Cantídeo. As características clínicas e as taxas de sucesso endoscópico do grupo pós-transplante hepático foram analisadas em comparação com as de outras etiologias.

**RESULTADOS:**

As principais causas de fístula biliar foram pós-transplante hepático (44%) e complicações da pós-colecistectomia (44%). O grupo pós-transplante hepático apresentou proporção significativamente maior de sexo masculino (pós-transplante hepático=81,8%, outros=28,6%) e idade mais avançada (pós-transplante hepático=54,1 anos, outros=42,0 anos) e maior incidência de estenose biliar (pós-transplante hepático=90,9%, outros=14,3%) do que o grupo com outras etiologias (p<0,05). Os dois grupos receberam tipos de tratamento semelhantes, dentre os quais a esfincterotomia associada à aposição de prótese biliar foi a mais utilizada. As taxas de sucesso terapêutico endoscópico não mostraram diferença significativa entre o grupo pós-transplante hepático (63,6%) e o grupo com outras etiologias (71,4%).

**CONCLUSÕES:**

O tratamento endoscópico das fístulas biliares secundária ao transplante hepático apresentou taxa de recuperação semelhante à de outras etiologias, apesar da idade avançada dos pacientes e da presença de estenose biliar.

## INTRODUCTION

Biliary fistulas typically occur as surgical complications, after laparoscopic cholecystectomy, liver transplantation (LT), or partial liver resection^9^. Most bile fistulas are not detected during surgical interventions and are only discovered after a significant delay, due to their nonspecific clinical presentation^6^ ^,^ ^23^; however, rapid clinical deterioration can occur due to peritonitis and sepsis. Therefore, early diagnosis and prompt treatment are important^22^. While the most common cause is laparoscopic cholecystectomy with an incidence of only 1%, post-LT biliary complications may also occur, with an incidence of 5-30%^1^ ^,^ ^21^. The incidence of bile duct injury following liver resection ranges from 2 to 25%^5^ ^,^ ^21^, whereas trauma is a particularly rare cause of bile duct injury, occurring in approximately 0.1% of patients with multiple trauma^14^.

Bile fistula is the second most common biliary adverse event after LT and is associated with significant morbidity in LT recipientes^13^. In addition, it is considered an independent risk factor for the development of early or late anastomotic biliary strictures and thus requires prompt, safe, and highly effective therapy^9^ ^,^ ^10^. While most bile fistulas occur within the first 30 days post-transplant and are related to technical issues at the anastomosis^20^, nonanastomotic fistula may also occur from the cystic duct stump or minor ducts, including the duct of Luschka^5^. Biliary strictures occur in approximately 12.8% of patients following LT^2^ ^,^ ^11^ and anastomotic strictures are most common, accounting for 80% of strictures^4^. Bile fistula at the anastomosis also predisposes patients to subsequent stricture formation through local inflammation and fibrosis^5^ ^,^ ^18^. In addition, the presence of a post-cholecystectomy bile fistula is in itself a risk factor associated with postoperative biliary stricture, with a reported incidence of 10-70% in a selected series^3^.

The management of biliary fistula requires a multidisciplinary approach involving hepatobiliary surgeons, interventional radiologists, and endoscopists. The first-line approach is endoscopic retrograde cholangiopancreatography (ERCP), which can be achieved through a variety of endoscopic techniques of which biliary sphincterotomy or biliary stenting or a combination of both is most widely used^22^. However, in the case of bile fistula following LT, the most widely accepted treatment in patients with a duct-to-duct biliary anastomosis is early ERCP. ERCP for bile fistula can be performed using either a combination of biliary sphincterotomy and plastic stent placement or sphincterotomy alone. Some authors propose the use of biliary sphincterotomy alone as it is easy to perform and patients do not require subsequent ERCP for stent removal; however, most of the available data supporting this practice was obtained from patients with bile fistula following cholecystectomy^19^.

Few studies have compared the success of endoscopic treatment, associated factors, and types of treatment between bile fistula related to LT and other etiologies; most medical literature has approached the subject in a sectional manner.

The primary objective of this study was to determine the efficacy of endoscopic treatment for bile fistula secondary to LT, compared with other etiologies. The secondary objective was to compare ERCP approaches to biliary fistula, the presence of biliary dilation/stenosis, and the need for additional surgery.

## METHODS

This retrospective study was performed in the endoscopic unit of the University Hospital Walter Cantídeo, Universidade Federal do Ceará (HUWC-UFC), a quaternary teaching hospital with a large number of hepatic transplant performed annually. It is important to note that all LTs performed at HUWC-UFC are from nonliving donors. All ERCPs performed in the hospital between 2015 and 2021 were also analyzed. All procedures were performed by an endoscopy resident under the supervision of an experienced interventional endoscopist, and deep sedation or general anesthesia was used depending on the patient’s status. This study was approved in the Local Ethics Committee (number: 43321014.6.0000.5045).

Among the 724 cases analyzed, 36 had a final diagnosis of “bile fistula.” Of these 36 cases, 11 cases of spontaneous suprapapillary fistula were excluded; therefore, the remaining 25 cases were analyzed. Data collected included age, sex, etiology, localization of the leak, primary catheterization, biliary dilatation, association with biliary stenosis, and if the endoscopy treatment was therapeutic (i.e., no need for additional surgery).

The etiologies of the fistula were divided into two groups: post-LT and other etiologies (including cholecystectomy, hepatectomy, and hepatic trauma). The locations of the fistula were divided into four major groups: cystic duct stump, common bile duct, hepatic common duct, and hepatic bed. Endoscopic therapeutic success was defined when there was no need for further surgery or radiological intervention after ERCP. The types of endoscopic therapy used in these cases were analyzed and divided into two groups: sphincterotomy associated with biliary stent apposition and isolated sphincterotomy; the choice of therapy was made by an endoscopist.

Data were analyzed using the GraphPad Prism software, and all tests were realized with a statistical significance of 5% (p=0.05). To compare the relevance of the statistics between the clinical variables for biliary fistula associated with LT versus other etiologies, we applied a 2×2 table using Fisher’s exact test and Student’s t-test.

## RESULTS

Among the 25 patients with bile fistula, 52% (13) were men, with a mean age of 47.3 (range, 16-71) years. Table 1 shows that LT was the etiology of the fistula in 44% (11) of patients, followed by cholecystectomy (44% [11]), hepatectomy (8% [2]), and hepatic trauma (4% [1]). Primary catheterization of the papilla was possible in 80% (n=20) of cases; of the remaining cases, fistulotomy (infundibulotomy) was successfully performed in 8% (n=2) and papillotomy in 12% (n=3). The most frequent location of the fistula was the post-LT anastomosis (36% [9]), followed by the cystic duct (32% [8]), common bile duct (16% [4]), common hepatic duct (4% [1]), and hepatic bed (12% [3]). The most frequent location of the leak after LT was the biliary anastomosis (82% [9]), followed by the cystic duct and common bile duct. After cholecystectomy, the most frequent location was the cystic duct stump (64% [7]), followed by the common bile duct (27% [3]) and common hepatic duct (10% [1]). In all cases secondary to hepatectomy (n=2) and liver trauma (n=1), the location of the leak was the liver bed.

**Table 1 -:** Etiology, localization, endoscopic findings, and treatment of the biliary fistula.

	n	%
Etiology of the leak		
Post-cholecystectomy	11	44
Post-liver transplantation	11	44
Hepatectomy	2	8
Hepatic trauma	1	4
Location of the fistula		
Cystic duct	8	32
Common bile duct	4	16
Common hepatic duct	1	4
Post-liver transplantation anastomosis	9	36
Hepatic bed	3	12
Primary catheterization	20	80
Biliary dilatation	15	60
Biliary stenosis	12	48
Therapeutic endoscopy	17	68

Table 1 demonstrates that 60% (15) of patients had biliary dilatation, while 48% (12) had biliary stenosis. Endoscopic treatment was effective in 68% (17) of patients; among them, the most prevalent approach was sphincterotomy and biliary stent placement (76% [13]), followed by isolated sphincterotomy (24% [4]). Among patients without endoscopic resolution (32% [8]), no endoscopic procedures were possible in half of the cases (4); in the other half, sphincterotomy and biliary stent placement were ineffective.

Table 2 shows that LT was the etiology in 44% (n=11) of cases, while other etiologies were responsible for 56% (n=14) of cases. While most patients in the post-LT group were male (81.8%), they represented only 28.6% of cases with other etiologies (p=0.02). Age varied between groups, with a higher mean observed in the post-LT biliary fistula group (54.1±4.4 years) than for other etiologies (42.0±4.6 years) (p=0.04). The presence of biliary tract dilatation associated with biliary fistula was verified in 54.2% of patients in the post-LT group versus 64.2% in the group with other etiologies (p>0.05). The presence of stenosis associated with biliary fistula was more pronounced in the post-LT group, where 90.9% of cases were associated with stenosis; among other etiologies, the presence of stenosis was identified in very few cases (14.3%) (p=0.001).

**Table 2 -:** Comparative results of the clinical characteristics of bile fistula secondary to post-liver transplantation compared to other etiologies.

	Post-liver transplantation n=11	Other etiologies n=14	p-value
Men	81.8%	28.6%	0.02*
Age (years)	54.1±4.4	42.0±4.6	0.04*
Biliary dilatation	54.5%	64.2	0.70
Biliary stenosis	90.9%	14.3%	0.001*
Primary catheterization	90.9%	50.0%	0.04*
Sphincterotomy and biliary stent	63.6%	71.4%	1.00

Data were analyzed by Fisher’s exact test and Student’s t-test.

Regarding primary catheterization, greater success was observed in the post-LT group, which demonstrated a success rate 90.9%; in the group with other etiologies, primary catheterization was only successful in half of the cases (p=0.04). The most prevalent type of treatment among all groups was sphincterotomy associated with biliary stent apposition, accounting for 63.6% of cases in the post-LT group and 71.4% in the group with other etiologies (p>0.05).

**Figure 1 -:**
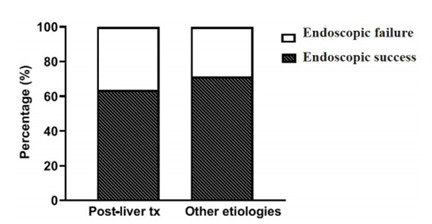
Therapeutic success of bile fistula secondary to post-liver transplantation compared to other etiologies (Fisher’s exact test and Student’s t-test).

Figure 1 demonstrates that therapeutic success, which is defined as resolution of the fistula by ERCP without the need for new surgeries or radiological interventions, exhibited few variations between groups. In the post-LT group, endoscopic success was achieved in 63.6% (7/11) of cases, whereas in the group with other etiologies, the endoscopic success rate was slightly higher at 71.4% (10/14) (p>0.05). It should be noted that, considering only the cases in which the primary catheterization was successful, the overall therapeutic success rate of ERCP for biliary fistula, regardless of etiology, was 85% (17/20).

## DISCUSSION

ERCP has been established as the best therapeutic option for biliary fistula following LT. Ross et al. stated that biliary leakage after duct-to-duct anastomosis is almost always managed endoscopically. Typically, insertion of a plastic stent with bridging of the anastomosis and leakage site is an adequate treatment; sphincterotomy is not necessary for this, and its risks should be balanced with its benefits (e.g., after difficult cannulation). Still, the optimal duration of endoscopic therapy remains unclear. In most centers, stents are removed via ERCP and repeated cholangiography after 6-8 weeks^15^. According to a recent meta-analysis, the combination of sphincterotomy and stenting was only preferred when the leak could be bridged. Thus, whether the placement of a bridging stent should be combined with sphincterotomy needs to be evaluated on a case-by-case basis, considering factors such as the risk of pancreatitis or bleeding. When considered safe, this combination appears to be the best choice. If the fistula cannot be bridged, stenting alone using a short stent may be the preferred option, as sphincterotomy alone, or a tab combining the short stent with a sphincterotomy, does not seem to improve the success rate. Furthermore, sphincterotomy-related complications can also increase morbidity. Surprisingly, most complications occurred in the stent placement group^22^.

Vlaemynck et al., in a recent meta-analysis, concluded that the first-line treatment for biliary fistulas, which are typically caused by laparoscopic cholecystectomy, was ERCP, wherein different endoscopic techniques could be used. The most common treatment techniques are sphincterotomy, stenting, or a combination of both. The reported success rates of all these interventions was very high (>90%), with no statistically significant differences between them^22^.

This study analyzed the efficacy of endoscopic treatment for bile fistula secondary to LT, compared with other etiologies. In addition, ERCP approaches to biliary fistula, the presence of biliary dilation/stenosis, and the need for additional surgery were also compared. The results demonstrated that the success rate of endoscopic therapy in its different forms was similar for biliary fistulae related to LT and other etiologies.

Finally, only 25 patients were included and the main etiologies related to biliary fistulae treated in our hospital were LT and cholecystectomy, accounting for 88% of cases (44% each). Corresponding with the literature, trauma accounted for only 1 (4%) case, while hepatectomy accounted for 2 (8%) cases.

The site of bile leakage must be differentiated according to etiology. Rio-Tinto et al. reviewed that, in the case of post-cholecystectomy biliary fistulae, Strasberg type A lesions are responsible for up to 85% of all cases (75% of cystic duct stump fistula and 10% of Luschka’s duct fistula)^15^. Among the 25 patients, 11 cases of biliary fistula were verified after cholecystectomy; among these 11 patients, 63.6% (7/11) of the fistulae occurred in the cystic duct stump, 27.3% (3/11) in the common hepatic duct, and 9% (1/11) in the common hepatic duct.

There was a relationship between biliary fistula and biliary anastomosis stenosis after LT in 90.9% (10/11) of patients; the site of the fistula was the biliary anastomosis in 81.8% of cases. In the comparative analysis, there was a relationship between stenosis and biliary fistula in 14.3% (2/14) of patients with other etiologies (p=0.001). Regarding post-LT fistula, most fistulas occur on the site of the anastomosis and are followed by anastomotic strictures in 26% of reported cases in the literature^16^.

in a retrospective study, Sánchez et al. analyzed 70 patients who underwent liver donor transplantation. Among them, 29 patients were diagnosed with bile leakage, accounting for 41.4% of the cohort; the source of the leak was the anastomosis in 23 (79.3%) patients (18). Unfortunately, these data are not useful for this study because LTs performed in the HUWC-UFC are from nonliving donors. The disparity between the data of this study and the literature, regarding the presence of anastomotic stenosis associated with biliary fistulae, can be explained by the selection bias in this study; as the patients analyzed had already a diagnosis of biliary fistula, the data were not representative of all LTs performed at the our hospital; rather, it represents the group of patients who underwent ERCP for biliary fistulae^17^.

Corroborated by the literature, there was a disparity when comparing sex associated with the prevalence of biliary fistula. Regarding biliary fistula associated with LT, 81.8% (9/11) of the patients were male. Conversely, males accounted for 28.6% (4/14) among other etiologies (p=0.02). In a recent review, Nephew et al. found that the disparity in LT varies by region, ranging anywhere from 4.6 to 13.9%. The hypotheses for the causes of this gender disparity include limitations on the ability of creatinine and therefore the MELD score to accurately predict renal function in women, donor-recipient size mismatch and difficulty finding appropriately sized organs for smaller statured women, and a lower likelihood of receiving hepatocellular carcinoma exception points. In addition, females are known to have a higher prevalence of cholestatic liver diseases, such as primary biliary cholangitis, than men, which may be more poorly aided by the MELD score than viral and alcohol-associated liver disease^12^.

In this study, males represented 81.8% (p=0.02), with a mean age of 54.1±4.4 years (p=0.04). In a 2018 review, Sendino et al. analyzed the data of 80 patients undergoing ERCP for biliary fistula associated with LT and reported data similar to this study finding^19^. Post-LT biliary fistulae were more prevalent in males (72.5 vs. 22.5%) and the average age was 54.7±10.3 years. In relation to other etiologies, the most prevalent in our study was post-cholecystectomy biliary fistula (44%, 11/25) and the lower prevalence of males has been described in the medical literature. The male-to-female ratio for age-adjusted cholelithiasis was reported to be 2.9 in patients aged 30-39 years and 1.2 in patients aged 50-59 years^8^.

Bile duct dilatation was observed in 54.5% of biliary fistula cases associated with LT and in 64.2% of cases related to other etiologies (p<0.05). Flumignan et al. reported different data in their analyses and found that the fistula-related biliary dilation was 25.8%. However, there are no data in the literature that associate fistula with the presence or absence of biliary tract dilatation. The conflicting data in relation to the aforementioned study may be related to the etiology; however, there were no biliary fistula associated with LT^7^.

In this study, the sphincterotomy followed by biliary stent apposition was the primary endoscopic therapy performed in 63.6% of patients with biliary fistula associated with LT, versus 71.4% in patients with other etiologies (p>0.05). Regardless of the relationship between biliary tract dilatation and bile duct stenosis, the difference in the success rate between groups was negligible. In the LT-associated biliary fistula group, the success rate of endoscopic therapy was 63.6% (7/11). Conversely, the success rate of the group with other etiologies was 71.4% (10/14) (p>0.05). However, considering only the cases in which the primary catheterization was successful, the overall therapeutic success rate of ERCP for biliary fistulas, regardless of etiology, was 85% (17/20) in the study period.

Regarding bile fistula after LT, available data regarding the use of biliary sphincterotomy alone are scarce and limited to a large series of patients treated for an array of adverse biliary events after LT. The success rate of this approach after LT is poorly understood, as no randomized controlled trials have directly compared this strategy to sphincterotomy plus biliary plastic stent placement. Sendino et al*.* reported that plastic stent placement for bile fistula after LT has the advantage of preferentially diverting bile flow to the duodenum by eliminating the transpapillary pressure gradient and could perhaps be why stent placement was responsible for better outcomes when compared with sphincterotomy alone^19^.

## CONCLUSION

Despite associated factors, such as biliary stenosis, sex, association with biliary tract dilatation, and location of the fistula, the success rate of endoscopic therapy in its different forms was similar, whether for biliary fistulae related to LT or fistula related to other etiologies, in this retrospective study.
